# Arterial–Venous Balance in the Human Renal Vasculature: Renal–Aortic and Renal–Caval Ratios and Factors on Contrast-Enhanced Computed Tomography

**DOI:** 10.3390/jcm15176852

**Published:** 2026-09-04

**Authors:** Grzegorz Wróbel, Tadeusz Kuder, Agnieszka Radowicz-Chil, Kinga Pirsztuk, Ryszard Maciejewski, Piotr Lewitowicz, Olga Adamczyk-Gruszka

**Affiliations:** 1 Department of Clinical and Experimental Pathology, Institute of Medical Sciences, Jan Kochanowski University, 25-516 Kielce, Poland; 2 Department of Anatomy, Institute of Medical Sciences, Jan Kochanowski University, 25-516 Kielce, Poland; 3 Department of Pathology, Holy Cross Cancer Centre, 25-734 Kielce, Poland; 4 Department of Plastic, Reconstructive and Hand Surgery, National Medical Institute of the Ministry of the Interior and Administration, 02-507 Warsaw, Poland; 5 Department of Surgical Clinical Sciences, Institute of Medical Sciences, Faculty of Medicine, The John Paul II Catholic University of Lublin, 20-708 Lublin, Poland; 6 Department of Gynaecology and Obstetrics, Institute of Medical Sciences, Jan Kochanowski University, 25-516 Kielce, Poland

**Keywords:** renal artery, renal veins, tomography, X-ray computed, contrast media, vascular morphometry, kidney transplantation, anatomic variation, clinical anatomy

## Abstract

**Background/Objectives**: A single absolute vessel diameter is hard to compare between individuals of differing body size. We defined two venous proportional parameters, the renal–caval ratio (R-IVCr) and factor (R-IVC_f_), as counterparts of the established renal–aortic ratio (R-Ar) and factor (R-A_f_), and asked whether the arterial and the venous bed respond to multiplicity of vessels in the same direction. **Methods**: Biphasic contrast-enhanced computed tomography of the abdomen in 1196 patients (2392 kidneys) was analysed retrospectively. Ratios related the dominant vessel diameter to that of the parent trunk; factors related the summed cross-sectional area of a kidney’s vessels to that of the corresponding trunk segment. Two angles, measured towards the left (α) and the right (β) from an anteroposterior reference axis, were recorded at both trunks. **Results**: Mean R-Ar was 0.2381 (±0.0573) and mean R-IVCr was 0.4651 (±0.0943). Dominant-vessel ratios were lower in the multiple variant and area-summing factors were higher, in parallel on both sides (*p* < 0.001). All four were higher in men, with moderate effect sizes arterially and negligible ones venously. α and β inverted between the two trunks, which was the largest effect recorded, and multiple veins showed a 2.3-fold right-sided predominance. **Conclusions**: The four parameters describe arterial inflow and venous outflow within a single proportional system in which both beds respond to multiplicity in parallel; their value in donor-kidney assessment and anastomosis planning awaits prospective validation.

## 1. Introduction

The renal arteries and veins are among the most variable elements of retroperitoneal topography. The classical description, a pair of single arteries arising from the abdominal aorta (AA) almost perpendicularly at the level of the disc between the first and second lumbar vertebrae, with corresponding veins draining into the inferior vena cava (IVC), represents only the most frequent of many arrangements [1]. The scale of departure from it can be striking: in a cadaveric series of 112 kidneys, a normal arrangement of the hilar structures was found in only 16% of organs, whereas the remainder displayed one of eight distinct variants [2]. The number, calibre and course of the vessels determine the conditions of parenchymal perfusion and, in clinical practice, bear on the selection of a kidney for procurement, on the course of denervation in resistant hypertension and on the safety of extensive abdominal interventions. These are decisions taken preoperatively on the basis of a contrast-enhanced computed tomography examination that is already part of routine clinical assessment, so the question addressed here is how that examination should be read.

An assessment of vascular supply based solely on the absolute diameter of a vessel meets an important limitation: the diameter of the renal artery is not an independent quantity. It remains coupled to the calibre of the aorta, and in individuals of greater body size, absolute values rise proportionally, obscuring differences that are genuinely morphological. For this reason, Majos et al. [3] proposed the renal–aortic ratio (R-Ar), the diameter of the dominant renal artery divided by the diameter of the aorta, as a relative measure robust to differences in body size. In the same study, they introduced the renal–aortic factor (R-A_f_), in which the summed cross-sectional areas of all arteries supplying a kidney are set against the cross-sectional area of the corresponding aortic segment; this construction accommodates the circumstance that a multiply vascularised kidney may be supplied by several vessels of smaller calibre. The idea of a relative index comparing the calibre of a branch with that of its parent trunk has recently been developed in other areas of vascular anatomy as well, an example being the δ index introduced for the deep femoral artery, which revealed a distinct sexual dimorphism in the proportional contribution of the branching vessel [4].

The venous bed has been described disproportionately less fully than the arterial one; however, it is the veins that are more often exposed to iatrogenic injury during dissection of the iliac vessels or during transplantation [5,6]. No parameter has so far captured venous outflow in a manner analogous to R-Ar and R-A_f_. The two parameters introduced here serve that purpose: the renal–caval ratio (R-IVCr), which relates the diameter of the dominant renal vein to the diameter of the IVC, and the renal–caval factor (R-IVC_f_), which sets the summed cross-sectional area of the veins draining a kidney against the cross-sectional area of the corresponding segment of the inferior vena cava. To our knowledge, this is the first description of both sides of the renal circulation within a single proportional framework.

The same imaging material has previously been analysed by our group in a separate study restricted to the numbers of multiple renal arteries and veins and their mutual correlation; that analysis included none of the parameters, angles or levels reported here, and the two datasets overlap only in the underlying examinations. The present work therefore addresses a different question. Do renal arterial inflow and venous outflow, expressed in the common language of relative parameters, follow parallel proportional patterns, and does multiplicity of vessels transform both sides of the circulation in a convergent way? Our working hypothesis was that the multiple variant, while reducing the dominance of a single trunk, simultaneously increases the total cross-sectional area, and that this pattern is repeated symmetrically on the arterial and the venous side. To anchor the parameters in topographical reality, the angles measured at both parent trunks and the levels at which the vessels occur relative to fixed reference points were included in the analysis. The aim of the study was accordingly threefold: to define R-IVCr and R-IVC_f_ as venous counterparts of the established arterial parameters; to describe the distribution of all four, together with the angles and craniocaudal levels of the vessels, by vascular variant, sex and body side; and to determine whether the arterial and the venous bed respond to multiplicity of vessels in the same direction. The design is anatomical and radiological. The examinations were performed for unrelated clinical indications in patients who were not candidates for kidney donation, and no operative or graft-outcome data were available, so the clinical applications considered in the Discussion are proposed rather than demonstrated.

## 2. Materials and Methods

### 2.1. Study Group

The material consisted of consecutive biphasic contrast-enhanced computed tomography (CT) examinations of the abdomen, obtained from 1196 patients investigated at the Department of Diagnostic Imaging of the Holy Cross Cancer Centre in Kielce, Poland. The group comprised 592 women and 604 men aged 19 to 92 years; mean age was 58.78 ± 15.31 years and differed significantly between the sexes (men 57.18 ± 14.74; women 60.41 ± 15.72; *p* = 0.0001). The primary set comprised 52,954 CT examinations performed between 2017 and 2019, of which 31,957 covered the abdomen and pelvis; after the criterion of at least biphasic acquisition was applied, 2751 patients remained, and after exclusions, 1196. No further sampling was applied at any stage: every examination that satisfied the criteria was measured.

Exclusion criteria were previous nephrectomy, developmental anomalies of the kidney (agenesis, hypoplasia, horseshoe kidney), a contracted kidney, pathological changes precluding reliable assessment of the vessels (large retroperitoneal or renal tumours), abdominal aortic aneurysm, and examinations of quality insufficient for precise measurement. The study was approved by the Bioethics Committee of the Faculty of Medicine and Health Sciences, Jan Kochanowski University in Kielce (Resolution No. 1/2019). All measurements were performed under the supervision of a specialist radiologist.

### 2.2. Imaging Protocol and Tools

Images in DICOM format were acquired with a 64-row Siemens SOMATOM Definition AS scanner (Siemens Healthineers AG, Erlangen, Germany). A non-ionic, low-osmolar iodinated contrast medium (Omnipaque 350, iohexol, 350 mg I/mL; GE HealthCare AS, Oslo, Norway) was administered intravenously with a dual-head power injector (Stellant CT; Bayer HealthCare LLC, Whippany, NJ, USA). Analysis was carried out on Siemens syngo Multi-Modality Workplace (syngo MMWP, software version VE40B) and Leonardo (syngo software version VE40B) workstations (Siemens Healthineers AG, Erlangen, Germany) using multiplanar reconstruction (MPR), maximum intensity projection (MIP) and three-dimensional volume rendering.The arterial and venous phases were assigned from the pattern of vascular enhancement on the images themselves rather than from nominal acquisition delays, and every assignment was verified by a specialist radiologist, which allowed the two beds to be assessed separately. All examinations were performed on clinical indications, so the volume and the flow rate of the injection were not standardised for research purposes and were not analysed.

### 2.3. Morphometric and Topographical Variables

**Figure 1:**
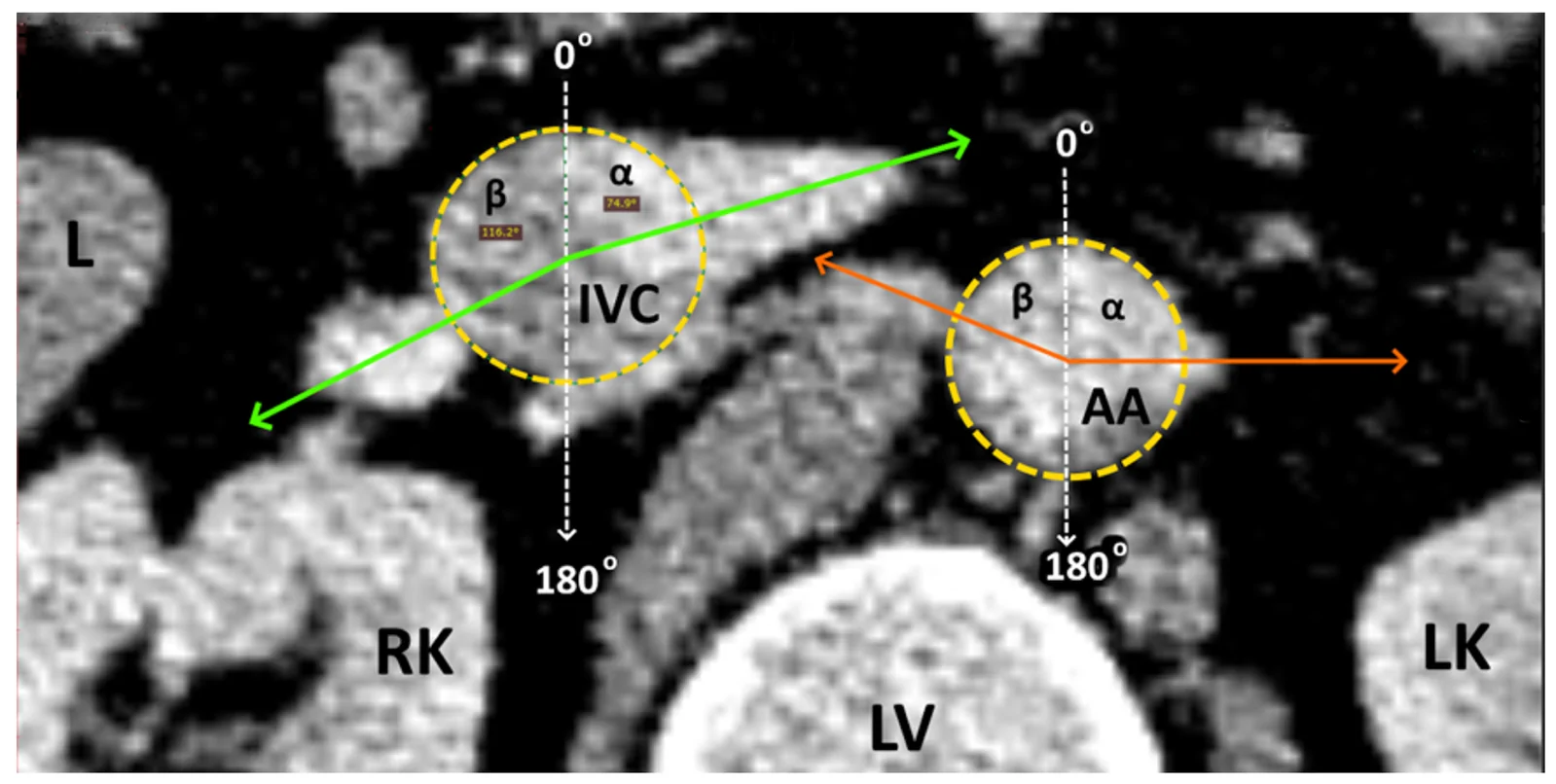
Principle of the angle measurement, illustrated in a transverse projection of contrast-enhanced computed tomography. At each parent trunk, an anteroposterior reference axis through the centre of the vessel is assigned 0° anteriorly and 180° posteriorly; the angle to the axis of the branching or terminating vessel is measured towards the patient’s left (α) and towards the patient’s right (β). Both angles are determined at the abdominal aorta (α, left renal artery; β, right renal artery) and at the inferior vena cava (α, left renal vein; β, right renal vein). α and β refer to two different vessels whose axes are not collinear, so their sum need not equal 180°; the double-headed arrows indicate the direction of each vessel and not a single continuous axis. Each angle was measured on a multiplanar reformation containing both the ostium and the point 15 mm distal to it, as set out in Section 2.3; that plane coincides with the native axial section only where the vessel runs purely transversely, so the projection reproduced here is illustrative. AA, abdominal aorta; IVC, inferior vena cava; L, liver; RK/LK, right/left kidney; LV, lumbar vertebral body; R/L, right/left side of the patient.

For each kidney, the number of arteries and veins was recorded, and the material was divided into single (sRA, sRV) and multiple (mRA, mRV) variants of vascularisation; throughout this paper, sRA and sRV denote the single-vessel variant, which differs from the convention of Majos et al. [3], in whom sRA denotes supernumerary renal arteries. The dominant vessel was the one of largest diameter; where two vessels of a kidney had identical diameters, the more cranial one was taken as dominant. Topography was captured by two angles measured at each parent trunk. A reference axis running through the centre of the trunk in the anteroposterior direction was assigned the values 0° anteriorly and 180° posteriorly; the angle between this axis and the axis of the branching or terminating vessel, taken 15 mm from the trunk, was then measured towards the patient’s left (α) and towards the patient’s right (β). Because the renal vessels frequently have a craniocaudal component to their course, the angle was read on a multiplanar reformation containing both the ostium and the point 15 mm distal to it, rather than on a single native axial section; this avoids the projection error that arises when an obliquely running vessel is measured in one transverse slice. Both angles were determined at the abdominal aorta, where α describes the left and β the right renal artery, and at the inferior vena cava, where α describes the left and β the right renal vein. The scheme for measuring the angles is shown in Figure 1.

**Figure 2:**
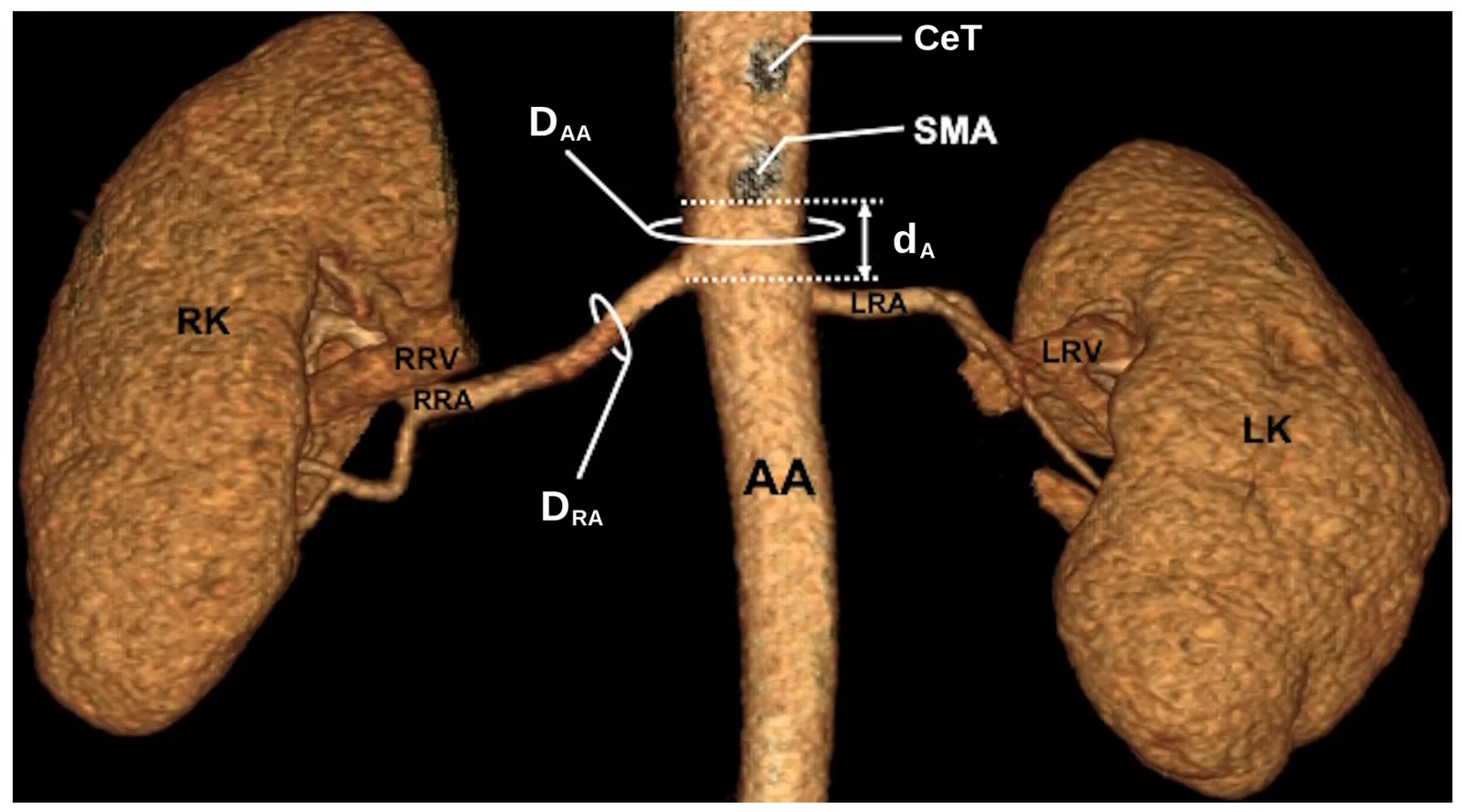
Scheme of the morphometric and topographical measurements, arterial side (three-dimensional reconstruction of contrast-enhanced computed tomography, arterial phase). Aortic diameter (D_AA_) measured 5 mm above the origin of the upper or single artery, renal artery diameter (D_RA_) 15 mm from the ostium, and craniocaudal distance of the origin from the SMA (d_A_). AA, abdominal aorta; SMA, superior mesenteric artery; CeT, coeliac trunk; RK/LK, right/left kidney; RRA/LRA, right/left renal artery; RRV/LRV, right/left renal vein.

The level at which the vessels occurred was expressed as the distance of the arteries from the superior mesenteric artery (SMA) and the distance of the veins from the confluence of the common iliac veins (cCIVs), both measured along the craniocaudal axis. The points at which diameters were measured were standardised as follows. The diameter of the aorta (D_AA_) was measured 5 mm above the origin of the upper or single artery, and the diameters of the renal arteries (D_RA_) 15 mm from their ostia (Figure 2).

**Figure 3:**
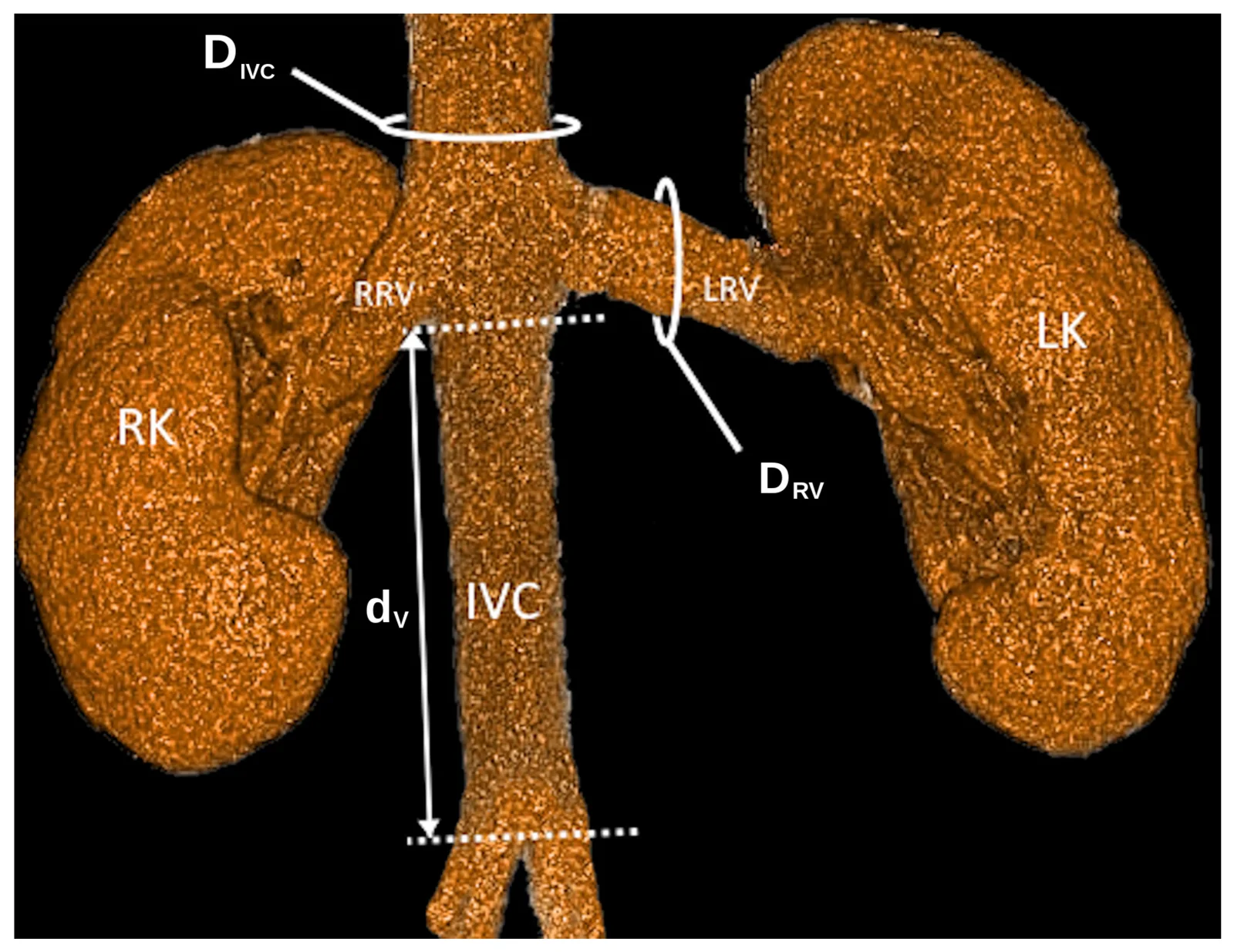
Scheme of the morphometric and topographical measurements, venous side (three-dimensional reconstruction of contrast-enhanced computed tomography, venous phase). Diameter of the inferior vena cava (D_IVC_), diameter of the renal vein (D_RV_) and craniocaudal distance of the termination from the confluence of the common iliac veins (d_V_). IVC, inferior vena cava; RK/LK, right/left kidney; RRV/LRV, right/left renal vein.

Analogously, the diameter of the inferior vena cava (DIVC) was recorded 5 mm above the termination of the highest or single vein, and the diameters of the renal veins (DRV) 15 mm from the IVC (Figure 3).

### 2.4. Definitions of the Parameters

The renal–aortic ratio (R-Ar) was defined as the diameter of the dominant renal artery divided by the diameter of the aorta, and the renal–caval ratio (R-IVCr) as the diameter of the dominant renal vein divided by the diameter of the IVC. Both ratios refer exclusively to the vessel of largest calibre. The factors, by contrast, describe the whole vascular bed of the kidney. The renal–aortic factor (R-A_f_) is the sum of the cross-sectional areas of all arteries supplying a given kidney divided by the cross-sectional area of the corresponding aortic segment; symmetrically, the renal–caval factor (R-IVC_f_) is the sum of the cross-sectional areas of all veins draining the kidney divided by the cross-sectional area of the corresponding segment of the inferior vena cava. The cross-sectional area of each vessel was derived from the measured diameter on the assumption of a circular lumen [π·(D/2)^2^]. Because each parameter sets a vessel against its own parent trunk measured in the same acquisition, in the same phase and at the same window setting, a difference between examinations in the degree of vascular opacification displaces numerator and denominator concordantly and is largely cancelled; this internal normalisation is the principal reason for preferring proportional to absolute measures here. By summing areas, the factor reflects the true areal contribution of inflow or outflow even when supply or drainage is distributed across several vessels of smaller calibre, which a ratio based on a single vessel cannot capture:

R-A_f_ = [π(D_RA1_/2)^2^ + … + π(D_RAn_/2)^2^]/π(D_AA_/2)^2^ (1)

R-IVC_f_ = [π(D_RV1_/2)^2^ + … + π(D_RVn_/2)^2^]/π(D_IVC_/2)^2^ (2)

where D_RA1_…D_RAn_ are the diameters of the successive arteries supplying a given kidney in Equation (1), D_RV1_…D_RVn_ are the diameters of the successive veins draining it in Equation (2), D_AA_ is the diameter of the aorta and D_IVC_ is the diameter of the inferior vena cava. R-Ar and R-A_f_ are adopted from Majos et al. [3]; R-IVCr and R-IVC_f_, their venous counterparts, are introduced in the present work.

### 2.5. Statistical Analysis

Calculations were performed in Statistica 12.0 (StatSoft, Tulsa, OK, USA). Conformity with the normal distribution was verified with the Shapiro–Wilk test. For normally distributed data, one- or two-way analysis of variance with Fisher’s least significant difference post hoc test was used, with homogeneity of variance assessed by the Levene and Brown–Forsythe tests and, where it was not met, by the Welch test. For distributions departing from normality, the Mann–Whitney U test or the Kruskal–Wallis test with Dunn’s multiple comparison test was applied; relationships between qualitative variables were examined with the chi-square test. Because the sample size makes trivially small differences statistically detectable, every rank-based comparison is reported together with the effect size r = |Z|/√*N*, interpreted according to the conventional thresholds of 0.1, 0.3 and 0.5 for small, moderate and large effects. Eighteen primary comparisons were performed, so a Bonferroni-adjusted threshold of α = 0.0028 was applied. Two further sensitivity analyses address the design. First, because the kidneys and vessels of one patient are not independent observations, the variance was inflated by a design effect of 1 + (m − 1)·ICC, where m is the mean number of observations per patient: 2.00 for the kidney-level parameters, 2.45 for the arterial and 2.31 for the venous vessel-level analyses; a conservative intraclass correlation coefficient (ICC) of 0.5 was assumed, and each test statistic was divided by the square root of the resulting design effect. Second, because the women in the cohort were on average 3.23 years older than the men and R-Ar is known to decline with age, the expected age-attributable component of the sex difference was estimated from the rank correlation of −0.36 reported by Majos et al. [3]. Neither analysis yields adjusted estimates or adjusted confidence intervals; both test how far the significance decisions depend on assumptions the design cannot satisfy. The intraclass correlation coefficient of 0.5 was chosen deliberately as an upper bound, since it assumes that half the total variance lies between patients, so the resulting inflation overstates rather than understates the penalty imposed by clustering. A generalised estimating equation or a mixed model with the patient as a random effect would be the appropriate treatment in a prospective design. The significance threshold was set at *p* < 0.05.

## 3. Results

A total of 2392 kidneys were included in the analysis of the arterial parameters (sRA, 78.34%; mRA, 21.66%) and 2390 kidneys in the analysis of the venous parameters (sRV, 86.53%; mRV, 13.47%); two kidneys whose veins drained into the common iliac vein were excluded from the venous analysis. The distribution of sex and body side remained almost even in both sets. The angles and levels were analysed at the level of the individual vessel, hence the larger counts (arteries, *n* = 2925; veins, *n* = 2760).

### 3.1. Renal–Aortic Ratio (R-Ar)

The mean R-Ar for the whole material was 0.2381 (±0.0573), with a range from 0.0951 to 0.5112. The single variant showed a higher value than the multiple variant (0.2437 versus 0.2178), and the difference was significant, with a small effect size (|Z| = 7.34; r = 0.15; *p* < 0.0001). A sexual dimorphism was also apparent: in men the ratio reached 0.2463 and in women 0.2297 (|Z| = 13.18; r = 0.27; *p* < 0.0001). The two effects were additive rather than interacting, the highest value being recorded in men with a single artery (0.2542) and the lowest in women with multiple arteries (0.2095); the full sex-by-variant breakdown is given in Table 1.

### 3.2. Renal–Aortic Factor (R-A_f_)

The factor R-A_f_ behaved inversely to the ratio based on the dominant vessel: its mean was higher in the multiple variant (0.1171) than in the single variant (0.0845), and this was the largest effect recorded in the study (|Z| = 23.35; r = 0.48; *p* < 0.0001). Men attained values exceeding those of women in both the sRA and the mRA group (|Z| = 14.16; r = 0.29; *p* < 0.0001) (Table 2). The comparison between variants indicates that the summed cross-sectional area of the arteries supplying a kidney does not decrease as the vascular bed becomes subdivided but, on the contrary, increases.

### 3.3. Renal–Caval Ratio (R-IVCr)

The mean R-IVCr for the whole group was 0.4651 (±0.0943), within a range of 0.2514–0.7731. The pattern revealed on the arterial side was repeated: the single variant exceeded the multiple variant (0.4696 versus 0.4353; |Z| = 7.45; r = 0.15; *p* < 0.0001), and the ratio was higher in men than in women (0.4721 versus 0.4574; |Z| = 3.28; r = 0.07; *p* = 0.0010) (Table 3). The venous values were at the same time almost twice as high as the arterial ones, which reflects the wider calibre of the veins relative to their parent trunk. The sex difference in R-IVCr carried the smallest effect size in the study and, together with the two angle comparisons noted in Section 3.5, was one of only three results that did not retain significance at the adjusted threshold once the variance was inflated by the design effect at ICC = 0.5.

### 3.4. Renal–Caval Factor (R-IVC_f_)

The symmetry between the two beds was expressed most fully in the behaviour of R-IVC_f_, which, like R-A_f_, proved higher in the multiple variant (0.2818) than in the single variant (0.2417; |Z| = 7.56; r = 0.15; *p* < 0.0001), with male values exceeding female ones in both groups (|Z| = 4.54; r = 0.09; *p* < 0.0001) (Table 4). Both sides of the circulation therefore responded to multiplicity of vessels in a convergent manner: with a fall in the dominance of a single trunk but a rise in the total cross-sectional area.

### 3.5. Angles at the Abdominal Aorta and at the Inferior Vena Cava (α and β)

At the abdominal aorta, the two angles differed markedly. The angle α, describing the left renal artery, approached a right angle (sRA: 94.13° ± 12.09°), whereas β, describing the right renal artery, was considerably more acute (sRA: 69.27° ± 12.57°). At the inferior vena cava, the relationship was reversed: α, for the left renal vein, was smaller (sRV: 74.99° ± 14.49°) and β, for the right renal vein, more obtuse (sRV: 116.24° ± 10.59°). The difference between α and β was highly significant at both trunks and carried a large effect size (aorta: |Z| = 30.35, r = 0.56; inferior vena cava: |Z| = 39.19, r = 0.75; *p* < 0.0001 in both cases). At the aorta, the anatomical variant (|Z| = 3.64; r = 0.07; *p* = 0.0003) and sex (|Z| = 3.59; r = 0.07; *p* = 0.0003) also reached significance, but with negligible effect sizes, and neither retained significance at the adjusted threshold once the design effect was applied; they should therefore be read as descriptive. Notably, in the multiple variant, the dispersion of values increased several-fold: the standard deviation of α reached 30.07° for mRA arteries and as much as 50.67° for mRV veins. Data for the aorta are collated in Table 5 and for the inferior vena cava in Table 6.

### 3.6. Level of the Vessels

The renal arteries arose from the aorta in close proximity to the superior mesenteric artery. In the single variant, the mean distance was 9.81 mm on the left and 7.60 mm on the right, and in the multiple variant, 15.86 mm and 12.33 mm respectively; the medians, however, were almost identical across variants (7.99 and 6.94 mm for sRA against 8.28 mm for mRA on both sides), so the difference in means is generated by a right-hand tail of low-origin accessory arteries rather than by a general displacement of the ostia. The same distinction applies to the veins. On the right, the shortening of the distance from the confluence of the common iliac veins in the multiple variant is present in both measures (median 111.52 mm for sRV against 98.21 mm for mRV), whereas on the left, it appears only in the means (117.77 against 97.90 mm) and disappears in the medians (117.45 against 114.98 mm), the median in the mRV group exceeding the mean, which indicates left skewness. The shortening of the venous distance in the multiple variant is therefore confined to the right side. All comparisons, by variant, by sex and by body side, were significant (*p* < 0.0001), with effect sizes ranging from small for the arterial distances (r = 0.11–0.19) to moderate for the variant effect on the venous distance (r = 0.38) (Table 7 and Table 8).

### 3.7. Side Asymmetry of the Multiple Variant

The side distribution of the multiple variant differed sharply between the two beds. Of the 1051 arteries of multiply vascularised kidneys, 573 (54.5%) supplied left and 478 (45.5%) right kidneys, a modest left-sided predominance. Of the 692 veins of the multiple variant, by contrast, 480 (69.4%) drained right and only 212 (30.6%) left kidneys, a 2.3-fold right-sided predominance. Expressed per organ, roughly two-thirds of multiply drained kidneys were right-sided, whereas multiple arteries were marginally more common on the left. The angles reflect the same asymmetry: the dispersion of α in the mRV group (SD 50.67°) is more than three times that of β (SD 15.01°), so the variability introduced by accessory veins is concentrated on the left side even though their number is not.

### 3.8. Arterial–Venous Balance

Setting the two beds within a single framework reveals a coherent pattern (Table 9). First, the parameters of relative dominance (R-Ar, R-IVCr) were systematically lower in the multiple variant, whereas the area-summing factors (R-A_f_, R-IVC_f_) were systematically higher; the direction of this relationship was identical on the arterial and the venous side, and the effect sizes for the two ratios were almost the same (r = 0.15 in both). Second, sexual dimorphism acted uniformly: men attained higher values of all four parameters; however, the effect was moderate on the arterial side (r = 0.27–0.29) and negligible on the venous side (r = 0.07–0.09). Third, venous values exceeded arterial ones almost twofold for the ratios and by about 2.4-fold in the multiple variant and 2.9-fold in the single variant for the factors, which reflects the greater capacity and wider calibre of the venous bed relative to arterial inflow.

## 4. Discussion

The contribution of the present work lies in treating four parameters as one internally coherent system describing both renal inflow and renal outflow. Our earlier analysis of the same material concentrated on the numbers of multiple vessels and their mutual correlation, considering them apart from any proportional assessment of the vascular bed. Here the emphasis falls on the relationship between the arterial and venous beds, read in the common language of relative parameters, and the perspective remains clinical, in that every parameter is derived from an examination already available before operation.

### 4.1. Embryological Background

Understanding vascular variability requires a return to organogenesis. On the classical ladder model, the definitive kidney, ascending from the sacral to the lumbar region, draws its supply successively from progressively higher segments of the aorta, while the more caudal mesonephric vessels regress, and persistence of any of them accounts for multiple arteries, including polar ones. Serial sections of human embryos have since shown that the mesonephric arteries are eliminated before glomeruli appear in the metanephros and that a symmetrical pair of renal arteries arises afterwards near its upper end, so the definitive vessel may not be a persisting segmental artery [7]. On either account, the number and level of the renal arteries are settled within this narrow window, and the variability recorded on imaging is its residue. The venous bed is formed differently, through anastomoses between the developing cardinal veins; persistence of these anastomoses may give rise to more than one renal vein and, in the extreme form, to a venous collar encircling the aorta. The report by Null et al. [8], describing the coexistence of a circumaortic and a retroaortic left renal vein in the same cadaver, illustrates how complex configurations may result from subtle disturbances in the regression of the embryonic venous arches. This difference in developmental mechanism accounts for the side asymmetry documented in Section 3.7. The right renal vein is the direct derivative of the right subcardinal system and is short, so supernumerary channels persist readily; the left renal vein is formed secondarily from the intersubcardinal anastomosis and must cross the midline, and it is the fate of that anastomosis, rather than the number of channels, that varies. Multiplicity is therefore more common on the right, whereas the variability of course, and hence of angle α, is concentrated on the left. Since development of the two beds proceeds in parallel, the convergent response of the arterial and venous parameters to multiplicity of vessels documented here appears to have a developmental rather than an incidental basis. The term parallel is used deliberately: the design permits no inference about functional coupling between inflow and outflow, and the concordance reported here is one of proportions.

### 4.2. Anatomical Variability and Its Associations Within the Renal Hilum

Majos et al. [3], on introducing R-Ar, demonstrated higher values in kidneys with a single supply than in multiply vascularised kidneys, an observation we confirm in full. A discrepancy concerns sex: those authors recorded higher R-Ar values in women, whereas in our considerably larger material, the direction of the difference was the reverse. Two explanations must be weighed before population difference is invoked. The first is age. Majos et al. reported a rank correlation of −0.36 between R-Ar and age, and in our cohort, the women were on average 3.23 years older than the men (*p* = 0.0001); applying that correlation, the age difference alone would depress the female mean by about 0.0044, which is roughly one quarter of the observed male–female gap of 0.0166 and leaves a residual sex effect of about 0.0122 in the same direction. The second is the composition of the cohorts: ours was drawn from an oncology referral centre and theirs from patients undergoing aortic CT angiography, and their study included 254 patients against our 1196. The reversal therefore appears to be attenuated but not abolished by age, and a formal age-adjusted model remains necessary before the difference is interpreted as biological. As for R-A_f_, Majos et al. [3] assumed that the summed cross-sectional areas of the arteries in the multiple variant should equal or exceed the value recorded in the single variant; our data support the second of these, with the largest effect size in the study (r = 0.48). The venous parameters behaved analogously, R-IVCr falling and R-IVC_f_ rising in the multiple variant, which extends the proportional description of the renal vascular bed to the outflow side, hitherto neglected.

Vascular variability should not, however, be considered in isolation from the remaining structures of the hilum. In the cadaveric material cited above [2], as many as 84% of kidneys showed a spatial arrangement of vein, artery and pelvis departing from the classical one, with a predominance of left-sided variants, an observation consistent with the asymmetry we observed in the angles and levels of the vessels. The multiplicity of proposed classifications of arterial and venous vascularisation [9,10,11,12,13] and the divergent data on the frequency of variants in successive populations [1,14,15,16,17,18,19,20,21,22] in fact expose the same difficulty that a relative parameter is intended to remedy: the absence of a common, quantifiable measure permitting comparison between organs of differing dimensions. One methodological point strengthens rather than weakens this finding. The aortic diameter used in the denominator of R-A_f_ was measured 5 mm above the origin of the upper or single artery and applied to all arteries of that kidney, yet the aorta tapers caudally and, as Table 7 shows, the ostia of accessory arteries are dispersed along the craniocaudal axis. For a low-lying accessory artery, the appropriate denominator would therefore be smaller than the one used, so R-A_f_ is systematically underestimated in the multiple variant. The bias acts against the direction of the observed difference, which nonetheless carried the largest effect size in the study (r = 0.48). The angles carry more than descriptive value. Because α and β were measured at both parent trunks against the same anteroposterior reference axis, the two beds can be compared directly, and the comparison shows an inversion: at the aorta, the left-sided angle is the larger (α > β), while at the inferior vena cava, it is the smaller (α < β). This inversion, with the largest effect sizes recorded in the study (r = 0.56 and 0.75), reflects the fact that the left renal artery leaves an aorta that lies to the left of the midline while the left renal vein must cross that midline to reach a vena cava lying to the right. Notably, in the multiple variant, the dispersion of values of both angles and levels sometimes increased several-fold, making the multiply vascularised kidney harder to anticipate intraoperatively. The lengthening of the distance of multiple arteries from the SMA and the shortening of the distance of multiple veins from the cCIVs further indicate that subdivision of the vascular bed is accompanied by dispersion of the points of origin along the craniocaudal axis, which can be a source of error when locating an accessory vessel [20,23].

### 4.3. Clinical Implications of the Parameters

The proposed four-parameter framework translates directly into three areas of clinical decision-making. First, into the qualification of a kidney donor: the higher areal factors in multiply vascularised kidneys call for revision of the view that multiplicity of vessels unconditionally impairs perfusion, since the summed cross-sectional area of the bed may in such cases be larger, albeit at the cost of the complexity of the anastomoses. Whether that geometry confers any functional advantage was not assessed here. Second, into the assessment of bleeding risk: venous outflow was relatively more abundant in men (higher R-IVCr and R-IVC_f_); however, the effect sizes for these sex differences were the smallest in the study (r = 0.07 and 0.09), and the available evidence on sex-related differences in the profile of haemorrhagic complications derives from percutaneous coronary intervention, where the higher risk was reported in women [24]. Any use of these parameters in bleeding-risk stratification therefore remains a hypothesis to be tested against surgical endpoints rather than a recommendation. Third, in the planning of vascular access, knowledge of the proportion of vessel calibre to parent trunk may be as important as the absolute value. Higher R-IVCr values describe a vein of larger calibre relative to its parent trunk. Whether this corresponds to more favourable outflow into the IVC was not tested, and any such reading would in addition require a normal venous course and the absence of compressive phenomena.

### 4.4. Relevance to Transplantation

Renal transplantation tests the proposed parameters most severely, since it brings together all the variables analysed, namely calibre, number, angle and level of origin of the vessels, and links them to measurable consequences. The very choice of the side of the donor organ may be dictated by morphometry: the left kidney is procured more often because of its significantly longer renal vein, which facilitates anastomosis. R-IVCr, which expresses the proportion of vein calibre to the inferior vena cava, offers a way of expressing this premise in proportional terms. None of the parameters reported here has been related to graft vascular complications, ischaemia time or graft survival, so the whole of this section is hypothesis-generating: the parameters are put forward as candidate descriptors for preoperative vascular characterisation, not as predictors of outcome.

The key transplantation dilemma, however, concerns multiply vascularised kidneys. Multiple arteries have long been reported to burden the graft with increased risk. A meta-analysis of kidney transplants with multiple renal arteries found significantly higher overall complication rates (13.8% versus 11.0%), more delayed graft function (10.3% versus 8.2%) and lower one-year graft survival (93.2% versus 94.5%), while five-year graft and patient survival were comparable [25]; a single-centre series of 451 deceased-donor transplants likewise showed warm ischaemia time rising significantly with the complexity of arterial reconstruction, without differences in delayed graft function or long-term graft survival [26]. Polar arteries pose a particular threat, since failure to account for them risks segmental infarction, urinary fistula and graft loss [9,15]. The scale of this multiplicity can be surprising: in our own observation, we documented a kidney supplied by as many as four separate arteries arising from the aorta on the left side [27], an extreme configuration in which reasoning about organ suitability from vessel number alone fails most acutely. Proportional assessment of the total cross-section is of most value in such cases. In this context, the areal factors (R-A_f_, R-IVC_f_) contribute information that the number of vessels alone does not provide: since they proved higher rather than lower in the multiple variant, the total cross-section of the vascular bed may be preserved, or even increased, despite subdivision into vessels of smaller calibre. This is consistent with the conclusion drawn from outcome data, that multiple donor renal arteries should not be regarded as a contraindication to transplantation [25,26], and suggests that the decision should rest on a proportional assessment of the total cross-section rather than on the arithmetic of vessels alone.

The practical usefulness of the topographical parameters emerges at the stage of back-table reconstruction and of the anastomosis itself. Because α and β are referred to a fixed anteroposterior axis rather than to the vessel itself, they state the spatial orientation of the renal vessel in the plane in which the surgeon meets it during dissection of the donor. They do not describe the geometry of the anastomosis, since the vessel is divided, mobilised and re-implanted in the iliac fossa, where its orientation is set by the recipient’s vasculature and by the operative technique. The inversion of the α–β relationship between aorta and vena cava nonetheless explains why right and left kidneys are approached differently at procurement. The level of origin, that is the distance of the arteries from the SMA and of the veins from the confluence of the common iliac veins, locates the ostium along the craniocaudal axis and so indicates where the vessel is to be sought and how far the aorta or the vena cava must be exposed. It is not equivalent to the length of vessel available for anastomosis, which depends on the distance from the ostium to the hilum, on the level at which the vessel is divided and on whether an aortic patch or a caval cuff is taken with it; none of these was measured here. Particular attention is demanded by the several-fold increase in the dispersion of angles and levels that we observed in the multiple variant: it means that an organ with accessory vessels is less predictable intraoperatively and that its procurement and implantation require more thorough preoperative planning.

This leads to a proposal rather than to a validated instrument: the four parameters, together with the angles and levels of origin, could be assembled into a standardised, CT-derived vascular passport of the donor kidney, a concise structured description of the vascular bed available before procurement. Whether such a description improves the decision on acceptance of the organ, on the choice of side or on the anticipated anastomotic technique is an empirical question that the present material cannot answer, and one that would require a prospective series in which the passport is recorded before procurement and related to ischaemia time, vascular complications and graft survival. Since it is the veins that are most often injured during dissection of the iliac vessels [5,6], prior knowledge of the relative calibre of venous outflow (R-IVCr, R-IVC_f_) may also prove useful, although this application awaits prospective validation.

### 4.5. Broader Surgical Context

Beyond transplantation, the parameters bear on a range of other operative pitfalls. An unrecognised circumaortic or retroaortic left renal vein constitutes a real risk of haemorrhage during retroperitoneal procedures and during repair of an abdominal aortic aneurysm [5,8], and its presence modifies the planning of portosystemic anastomoses [6]. A high, narrow course of the superior polar artery in turn risks inadvertent division and rapid bleeding. In each of these situations, the areal factor informs not only how many vessels supply or drain a kidney but also what proportion of the cross-section of the parent trunk they jointly represent, and hence the scale of potential blood loss. The proposed framework thus belongs to the current of clinically oriented vascular morphometry exemplified by the relative indices being developed for other regions of the arterial bed [4].

### 4.6. Limitations and Directions for Future Research

Several limitations qualify these findings. The study rests on single-centre material and is cross-sectional in design; it therefore permits no inference about causal relationships or functional consequences, and the cohort, drawn from an oncology referral centre, may not represent the general population. Measurements were performed by a single observer under the supervision of a specialist radiologist, and intra- and inter-observer reproducibility was not formally quantified; this is the principal methodological limitation, and prospective work should report intraclass correlation coefficients for every measured quantity. Measurement of diameters at strictly defined points, although reproducible, simplifies the true geometry of the vessels, and the areal factors assume a circular cross-section, which in veins may be flattened; the calibre of the inferior vena cava additionally depends on volaemic status and respiratory phase at the moment of acquisition, so both renal–caval parameters carry a physiological variability that the arterial parameters do not. The angles were read on reformations containing both the ostium and the point 15 mm distal to it, which removes the projection error of a single axial section but introduces a dependence on the operator’s choice of reformation plane that was not quantified. The two kidneys of one patient are not independent observations, and the angles and levels were analysed at the level of the individual vessel, so the effective sample size is smaller than the nominal one; in the sensitivity analysis described in Section 2.5, fifteen of the eighteen primary comparisons retained significance at the adjusted threshold, the exceptions being the sex difference in R-IVCr and the variant and sex effects on the angle at the aorta, which are the three results with the smallest effect sizes (r = 0.07); a generalised estimating equation or mixed model with the patient as a random effect would nonetheless be the appropriate treatment. Age was not adjusted for in the primary analysis, and the sensitivity calculation reported in Section 4.2 suggests that roughly a quarter of the sex difference in R-Ar may be age attributable. Body surface area was not available, so the claim that the parameters are independent of body size rests on their construction rather than on direct demonstration. A natural extension would be a prospective comparison of the four parameters, together with the angles and levels of origin, with the fate of the graft: ischaemia time, the frequency of vascular complications and graft survival, and also with direct measures of flow such as CT perfusion. It also appears promising to incorporate the parameters into machine-learning models that support donor qualification and to extend the analysis to associations with the variability of the remaining hilar structures, the scale of which, as the most recent cadaveric studies show [2], remains underestimated. Two further limitations bear on the interpretation of the areal factors and venous parameters. Summed cross-sectional area is not a proxy for flow: flow through a tube scales with the fourth power of the radius, so a bed subdivided into *n* vessels of equal calibre carrying the same total cross-section as one large vessel presents *n* times the resistance, and subdivision raises resistance even where total area is preserved or increased. The higher factors recorded in the multiple variant therefore describe the geometry of the bed and not its conductance. Cardiac function was not recorded either, and this matters asymmetrically. The ratios are internally normalised and largely cancel a systematic difference in opacification between examinations, but the inferior vena cava is a compliant vessel whose calibre reflects central venous pressure and volaemic status, so a raised right-sided filling pressure lowers R-IVCr and R-IVC_f_ without any change in the renal veins themselves; reduced cardiac output additionally prolongs bolus transit and may leave the renal veins incompletely opacified at a fixed acquisition delay. Prospective work should record cardiac status and trigger the venous phase by bolus tracking. The variables held in the anonymised database were age, sex, body side, the number of arteries and veins of each kidney and the measured diameters, angles and craniocaudal levels; anthropometric data, blood pressure, serum creatinine and estimated glomerular filtration rate, cardiac function data and the oncological diagnosis were not recorded in the radiological information system and could not be retrieved once the records had been anonymised. Body surface area, estimated glomerular filtration rate and, on the venous side, central venous pressure or an echocardiographic surrogate are the quantities that prospective work should record alongside the imaging, with warm and cold ischaemia time, delayed graft function, the frequency of vascular complications and graft survival as the outcome measures against which the parameters would have to be tested.

## 5. Conclusions

Four morphometric parameters, R-Ar, R-A_f_, R-IVCr and R-IVC_f_, form a coherent framework describing renal inflow and outflow within a single proportional system. Multiplicity of vessels lowers the dominance of a single trunk (lower R-Ar and R-IVCr) but raises the total cross-sectional area of the bed (higher R-A_f_ and R-IVC_f_), and this pattern is repeated in parallel on the arterial and venous sides. All four parameters were higher in men, although the effect was moderate on the arterial side and negligible on the venous side. The angles α and β, measured at both parent trunks against a common anteroposterior reference axis, invert between the aorta and the inferior vena cava and show the largest effect sizes in the study, while their dispersion increases markedly in the multiple variant. Multiplicity of veins showed a 2.3-fold right-sided predominance, whereas multiple arteries were marginally more common on the left, an asymmetry consistent with the different embryological origin of the two beds. The parameters are of greatest practical value in transplantation: they permit proportional assessment of the total cross-section of the vascular bed of a multiply vascularised kidney instead of disqualification of the organ on the basis of vessel number alone, and together with the angles and levels of origin, they may inform the choice of side and the planning of anastomoses. The functional and prognostic significance of the parameters was not examined here, and prospective validation against surgical endpoints is the necessary next step.

## Figures and Tables

**Table 1:** Values of the renal–aortic ratio (R-Ar) by anatomical variant and sex.

Category	Group	*N*	Mean	Median	Minimum	Maximum	SD
Overall		2392	0.2381	0.2200	0.0951	0.5112	0.0573
Variant	sRA	1874	0.2437	0.2226	0.1162	0.5112	0.0568
	mRA	518	0.2178	0.2104	0.0951	0.4239	0.0547
Sex	Men	1208	0.2463	0.2260	0.0951	0.5112	0.0569
	Women	1184	0.2297	0.2137	0.1187	0.5023	0.0566
Sex × variant	Men, sRA	898	0.2542	0.2311	0.1162	0.5112	0.0567
	Men, mRA	310	0.2235	0.2147	0.0951	0.4239	0.0512
	Women, sRA	976	0.2341	0.2144	0.1251	0.5023	0.0552
	Women, mRA	208	0.2095	0.2076	0.1187	0.3534	0.0587

R-Ar, renal–aortic ratio; SD, standard deviation; sRA/mRA, single/multiple variant of arterial vascularisation. The sex-by-variant rows show that the two effects act in the same direction without interaction. R-Ar is dimensionless.

**Table 2:** Values of the renal–aortic factor (R-A_f_) by anatomical variant and sex.

Variant	Group	*N*	Mean	Median	Minimum	Maximum	SD
sRA	Overall	1874	0.0845	0.0747	0.0307	0.3086	0.0339
	Men	898	0.0930	0.0885	0.0408	0.3086	0.0349
	Women	976	0.0766	0.0696	0.0307	0.2823	0.0309
mRA	Overall	518	0.1171	0.1102	0.0736	0.5906	0.0348
	Men	310	0.1219	0.1123	0.0841	0.5906	0.0393
	Women	208	0.1097	0.1087	0.0736	0.3814	0.0251

R-A_f_, renal–aortic factor (summed cross-sectional area of the arteries/cross-sectional area of the aorta); sRA/mRA, single/multiple variant of arterial vascularisation. R-A_f_ is dimensionless.

**Table 3:** Values of the renal–caval ratio (R-IVCr) by anatomical variant and sex.

Category	Group	*N*	Mean	Median	Minimum	Maximum	SD
Overall		2390	0.4651	0.4526	0.2514	0.7731	0.0943
Variant	sRV	2068	0.4696	0.4589	0.2539	0.7731	0.0963
	mRV	322	0.4353	0.4234	0.2514	0.6835	0.0737
Sex	Men	1208	0.4721	0.4584	0.2539	0.7731	0.1053
	Women	1182	0.4574	0.4507	0.2514	0.7446	0.0804

R-IVCr, renal–caval ratio; sRV/mRV, single/multiple variant of venous vascularisation. R-IVCr is dimensionless. The sex difference in R-IVCr did not retain significance at the adjusted threshold once the design effect described in Section 2.5 was applied and is therefore descriptive.

**Table 4:** Values of the renal–caval factor (R-IVC_f_) by anatomical variant and sex.

Variant	Group	*N*	Mean	Median	Minimum	Maximum	SD
sRV	Overall	2068	0.2417	0.2119	0.0798	0.5987	0.1005
	Men	1023	0.2583	0.2161	0.1072	0.5987	0.1193
	Women	1045	0.2254	0.2064	0.0798	0.5545	0.0742
mRV	Overall	322	0.2818	0.2747	0.1351	0.6951	0.1059
	Men	185	0.2913	0.2847	0.1351	0.6951	0.1179
	Women	137	0.2690	0.2642	0.1552	0.6231	0.0859

R-IVC_f_, renal–caval factor (summed cross-sectional area of the veins/cross-sectional area of the IVC); sRV/mRV, single/multiple variant of venous vascularisation. R-IVC_f_ is dimensionless.

**Table 5:** Angles of the renal arteries at the abdominal aorta by anatomical variant [°].

Variant	Angle	*N*	Mean	Median	Minimum	Maximum	SD
sRA	α (left)	916	94.13	91.68	58.63	134.00	12.09
	β (right)	958	69.27	71.23	31.25	97.39	12.57
mRA	α (left)	573	82.03	87.68	7.14	142.74	30.07
	β (right)	478	70.25	69.23	9.12	128.16	25.84

α and β, angles measured from the anteroposterior reference axis of the abdominal aorta (0° anterior, 180° posterior) towards the patient’s left (α, left renal artery) and right (β, right renal artery). *N* = 2925 arteries (sRA, 1874; mRA, 1051). Difference between α and β: |Z| = 30.35, r = 0.56; anatomical variant: |Z| = 3.64, r = 0.07; sex: |Z| = 3.59, r = 0.07; *p* ≤ 0.0003 in all cases. The two latter comparisons carried negligible effect sizes and did not retain significance at the adjusted threshold once the design effect was applied.

**Table 6:** Angles of the renal veins at the inferior vena cava by anatomical variant [°].

Variant	Angle	*N*	Mean	Median	Minimum	Maximum	SD
sRV	α (left)	1088	74.99	74.20	43.43	133.21	14.49
	β (right)	980	116.24	112.65	97.30	159.83	10.59
mRV	α (left)	212	86.09	45.25	29.37	169.12	50.67
	β (right)	480	113.22	110.21	78.21	171.05	15.01

α and β, angles measured from the anteroposterior reference axis of the inferior vena cava (0° anterior, 180° posterior) towards the patient’s left (α, left renal vein) and right (β, right renal vein). *N* = 2760 veins (sRV, 2068; mRV, 692). Difference between α and β in the sRV group: |Z| = 39.19; r = 0.75; *p* < 0.0001. The marked separation of median and mean for α in the mRV group (45.25° versus 86.09°, SD 50.67°) reflects a bimodal distribution, with accessory veins terminating either at a sharply acute or at an obtuse angle.

**Table 7:** Distance of the origin of the renal arteries from the superior mesenteric artery (SMA) by anatomical variant and body side [mm].

Variant	Side	*N*	Mean	Median	Minimum	Maximum	SD
sRA	Left	916	9.81	7.99	0.00	60.90	6.49
	Right	958	7.60	6.94	0.01	58.90	5.76
mRA	Left	573	15.86	8.28	0.01	139.00	21.65
	Right	478	12.33	8.28	0.01	129.00	17.88

Distance measured along the craniocaudal axis [mm]; *N* = 2925 arteries. Differences significant with respect to variant (|Z| = 6.67; r = 0.12), sex (|Z| = 5.88; r = 0.11) and body side (|Z| = 10.48; r = 0.19); *p* < 0.0001 in all cases. The considerable difference between mean and median in the multiple variant indicates right skewness of the distribution and the presence of extreme values.

**Table 8:** Distance of the termination of the renal veins from the confluence of the common iliac veins (cCIVs) by anatomical variant and body side [mm].

Variant	Side	*N*	Mean	Median	Minimum	Maximum	SD
sRV	Left	1088	117.77	117.45	88.39	142.39	8.91
	Right	980	112.41	111.52	83.23	137.52	8.56
mRV	Left	212	97.90	114.98	34.21	134.08	24.99
	Right	480	93.81	98.21	29.41	129.49	22.55

Distance measured along the craniocaudal axis [mm]; *N* = 2760 veins. Differences significant with respect to variant (|Z| = 19.96; r = 0.38), sex (|Z| = 4.13; r = 0.08) and body side (|Z| = 9.23; r = 0.18); *p* < 0.0001 in all cases.

**Table 9:** Synthetic summary of the four parameters: mean values by anatomical variant, with the test statistic and effect size for the variant comparison.

Parameter	Single Variant	Multiple Variant	|Z|	r
R-Ar	0.2437	0.2178	7.34	0.15
R-A_f_	0.0845	0.1171	23.35	0.48
R-IVCr	0.4696	0.4353	7.45	0.15
R-IVC_f_	0.2417	0.2818	7.56	0.15

|Z|, absolute value of the Mann–Whitney test statistic for the comparison between the single and the multiple variant; r = |Z|/√*N*, effect size; *p* < 0.0001 for all four comparisons. The corresponding sex comparisons were: R-Ar, |Z| = 13.18, r = 0.27; R-A_f_, |Z| = 14.16, r = 0.29; R-IVCr, |Z| = 3.28, r = 0.07; R-IVC_f_, |Z| = 4.54, r = 0.09.

## Data Availability

The anonymised measurement dataset underlying the results, together with the analysis script used to reproduce every table, is available from the corresponding author on reasonable request and will be deposited in an institutional repository upon acceptance. The source imaging examinations cannot be shared because they remain subject to patient-data protection.

## Abbreviations

AA, abdominal aorta; cCIVs, confluence of the common iliac veins; CT, computed tomography; d_A_, craniocaudal distance of the origin of a renal artery from the SMA; d_V_, craniocaudal distance of the termination of a renal vein from the cCIVs; D_AA_, diameter of the abdominal aorta; DICOM, Digital Imaging and Communications in Medicine; D_IVC_, diameter of the inferior vena cava; D_RA_, diameter of a renal artery; D_RV_, diameter of a renal vein; ICC, intraclass correlation coefficient; IVC, inferior vena cava; MIP, maximum intensity projection; MPR, multiplanar reconstruction; mRA/mRV, multiple renal arteries/veins; r, effect size |Z|/√*N*; R-A_f_, renal–aortic factor; R-Ar, renal–aortic ratio; R-IVC_f_, renal–caval factor; R-IVCr, renal–caval ratio; SD, standard deviation; SMA, superior mesenteric artery; sRA/sRV, single renal artery/vein.
